# Effectiveness of Preoperative Alpha Wave Entrainment in Pediatric Dental Patients: A Randomized Controlled Trial

**DOI:** 10.7759/cureus.60154

**Published:** 2024-05-12

**Authors:** Farah Shehani A, Victor Samuel A, Kavitha Ramar, Rekha Mani

**Affiliations:** 1 Pediatric Dentistry, SRM Kattankulathur Dental College, SRM Institute of Science & Technology, Chennai, IND; 2 Pedodontics and Preventive Dentistry, SRM Kattankulathur Dental College, Chennai, IND; 3 Endodontics, SRM Kattankulathur Dental College, SRM Institute of Science & Technology, Chennai, IND

**Keywords:** audio-visual distraction, binaural beats, non-pharmacological interventions, randomized control trial, dental fear and anxiety, fear and anxiety, vital signs, pediatric preventive dentistry, alpha waves, brainwave entrainment

## Abstract

Background

Pediatric dental anxiety is a significant barrier to effective dental care, necessitating non-pharmacological interventions. Alpha wave entrainment has shown promise in adult studies for reducing procedural anxiety and pain perception, but its effectiveness in pediatric dental settings remains underexplored.

Objective

This study aims to evaluate the effectiveness of preoperative alpha wave entrainment in alleviating anxiety in gender-specific participants to the interventions.

Methods

We conducted a randomized controlled trial involving 252 pediatric patients (aged 7-12) with cooperative dispositions. Participants were randomly assigned to either an experimental group receiving alpha wave entrainment or a control group receiving conventional behavior management techniques. The experimental intervention involved 10-minute sessions of binaural beats with visual stimulation designed to induce alpha-wave synchronization. Anxiety levels were assessed using physiological measures (heart rate and blood pressure), both pre- and post-interventions.

Results

The intervention group demonstrated a significant reduction in heart rate and systolic blood pressure post-intervention compared to the control group. These changes indicate a decrease in anxiety levels, with no significant gender differences in the response to the intervention.

Conclusion

Alpha wave entrainment effectively reduces dental anxiety in pediatric patients, with similar efficacy observed across genders. This study supports the incorporation of alpha wave entrainment into pediatric dental practices as a viable alternative to traditional anxiety management techniques.

## Introduction

The phenomenon of dental anxiety and discomfort within pediatric dentistry necessitates the exploration of innovative, non-pharmacological interventions aimed at ameliorating the treatment experience for pediatric patients. In this context, alpha wave entrainment emerges as a noteworthy intervention. This modality, characterized by the synchronization of cerebral wave frequencies with specific auditory or visual cues, has been documented to foster a state of relaxation and well-being. Empirical evidence from adult groups, as delineated by Udo et al., exhibits the significant potential of brainwave synchronization in attenuating procedural anxiety, positing a promising application in pediatric domains [1]. Additionally, Lopez et al. elucidated the utility of alpha wave entrainment in the efficacious management of pain perception, reinforcing its suitability for pediatric dental care applications [2].

Despite the promising results from research focused on adults, there is a need to further investigate the use of alpha wave entrainment to reduce dental anxiety in children. This group is particularly sensitive to dental procedures and may benefit differently from such strategies. This gap in the literature is particularly salient given the imperative challenges encountered by dental practitioners in navigating pediatric patients' anxiety, a factor that markedly influences the caliber of care administered. The seminal investigations conducted by Giani et al. demonstrated the efficacy of non-pharmacological interventions in significantly reducing dental anxiety among pediatric cohorts and advocated for the examination of alpha wave entrainment within this population [3,4].

The exigency for an exhaustive assessment of the effectiveness of alpha wave entrainment in pediatric dental contexts is further accentuated by the preliminary findings of Joyce et al., who reported positive implications of such interventions on pediatric patients' cooperative behavior during dental treatments [5,6]. These observations lay a foundational premise for the present study, which endeavors to methodically evaluate the influence of alpha wave entrainment on both male and female pediatric dental patients subjected to interventions.

Incorporating a rigorous methodological framework and leveraging empirical data from antecedent research, this study aims to substantiate the efficacy of alpha wave entrainment as a credible intervention for mitigating preoperative dental anxiety, whilst exploring the existence of potential gender-specific responses to the treatment modality. Hence, this investigation aspires to delineate a scientifically validated pathway for integrating anxiety-reducing techniques into pediatric dental practices, potentially heralding a paradigm shift in the management of dental anxiety and augmenting the therapeutic experience for pediatric patients.

## Materials and methods

Study design

A randomized controlled trial was conducted, and the study protocol received approval from the Institutional Ethical Committee under reference number 3010/IEC/2021. The study was subsequently officially registered in the Clinical Trial Registry of India (CTRI) database under the reference code CTRI/2023/03/051066.

**Figure 1:**
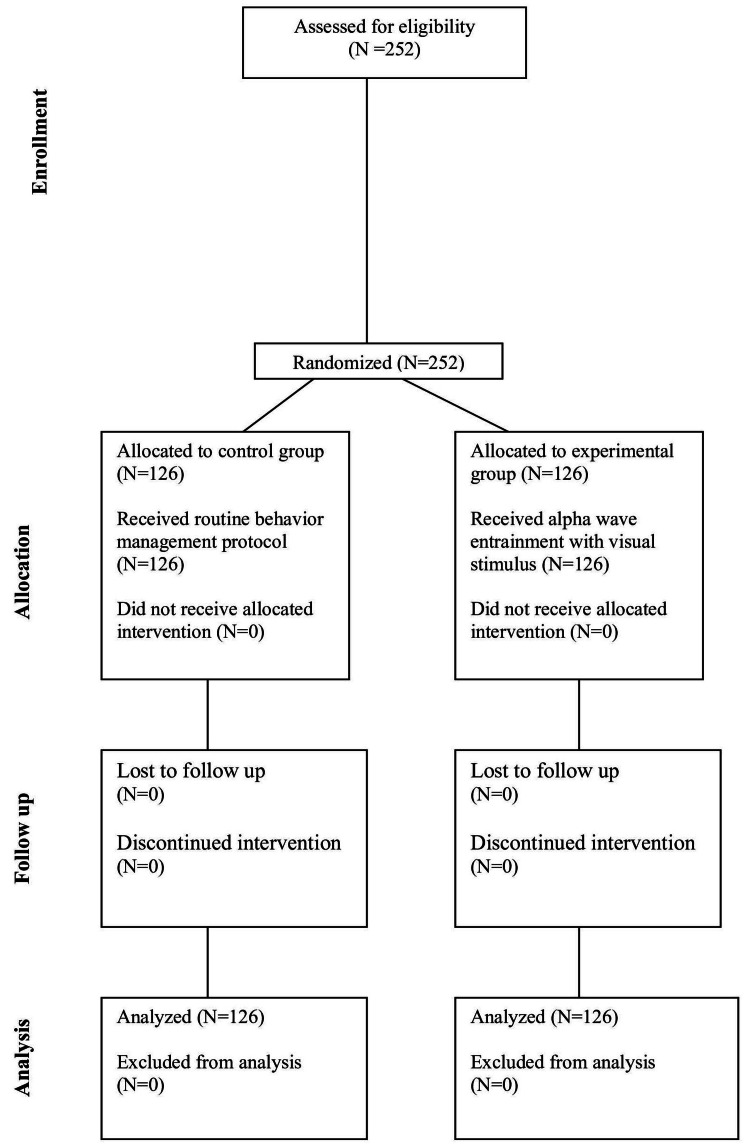
Consort flow chart. The data represented here: N- No. of participants.

Sample size estimation

The estimation of the required sample size for our study was conducted utilizing the G*Power software, applying a set of predefined parameters to ensure statistical robustness and sensitivity. These parameters included an anticipated effect size (Cohen's d) of 0.5, a significance level (α error probability) set at 0.05, and statistical power (1 - β error probability) aimed at 0.95. The application of these parameters yielded a requisite sample size of 252 individuals.

Eligibility criteria

Inclusion Parameters

This investigation targeted pediatric subjects aged 7 to 12 years, who were characterized by cooperative dispositions as evidenced by the favorable Frankl behavior scale assessments. Enrollment was confined to those receiving dental interventions within the pediatric dentistry division.

Exclusion Parameters

Exclusion criteria encompassed participants demonstrating non-cooperative behaviors that necessitated the application of additional behavioral management strategies during dental procedures. Moreover, the study precluded the participation of individuals with pre-existing medical conditions susceptible to influence or be influenced by brainwave entrainment modalities, such as neurological disorders, cerebral trauma, or sensory deficits. Consent refusal by either the prospective participants or their legal guardians also led to exclusion from the study group.

Randomization protocol

Utilizing the digital platform "randomizer.org," the study generated 126 dyads of unique numerical identifiers spanning from 1 to 2. After ensuring methodological integrity and blinding, participant details were encapsulated within opaque, sequentially allocated envelopes. This subsequently facilitated the equitable random assignment of 252 subjects into two groups: an experimental group and a control group. Group allocation remained concealed from the data analyst until the study's conclusion.

Informed consent process

Detailed elucidation of the study’s methodology was provided to both the pediatric participants and their guardians or parents. This included an introductory exposure to the specific audiovisual (AV) stimulation to be deployed. Formal consent was then procured in written form from guardians or parents, supplemented by verbal assent from the pediatric participants.

Intervention methodology

The experimental group was subjected to a binaural beat stimulus aiming to induce alpha wave (10 Hz) entrainment through differential auditory inputs (Left Stimulus: 9.5 Hz, Right Stimulus: 10.5 Hz), delivered via the "David Delight Plus Device Software" developed by Mind Alive, Inc., Alberta, Canada. This setup included compatible headsets and a multicolored eyeset for smartphone linkage. Contrastingly, the control group was exposed to conventional behavioral management techniques such as tell-show-do, tell-play-do, euphemisms, modeling, and distraction. Each intervention was conducted for 10 minutes.

Standardization measures

The standardized volume threshold was set at a sound pressure level of 60 dB, with provisions for participant-led adjustments to enhance comfort. Ambient clinical noise was minimized to obviate external auditory disturbances, and subjects were advised to maintain eye closure throughout the intervention duration. To mitigate potential bias, the principal investigator limited interactions with participants strictly to necessary procedural instructions.

Assessment

Prior to the intervention, a comprehensive psychophysiological evaluation of fear and anxiety manifestations was systematically undertaken, employing pulse rate and blood pressure as key physiological markers. This assessment was facilitated through the utilization of an automated blood pressure calibration device (OMRON, 5 Series, Model-BP7200), which featured an integrated display for immediate readouts. Baseline physiological measurements were duly recorded as HR1 (heart rate) and BP1 (blood pressure), serving as reference points against which post-intervention values, denoted as HR2 and BP2, were compared.

To uphold the integrity of the scoring and evaluation process, a single investigator maintaining a uniform methodological approach was tasked with the assessment of all participants. Following a temporal span of 10 minutes post the initial evaluation, the anxiety metrics of the subjects were reassessed, setting the stage for the subsequent dental interventions. In the aftermath of the intervention, the audio-visual equipment, comprising headphones and eye sets, was subjected to a rigorous sanitization protocol employing alcohol-based wipes to ensure hygiene and safety standards. 

Data collection

Data collected from physiological monitors were subsequently transferred to Microsoft Excel for initial organization and formatting.

Statistical analysis:

Heart rate and blood pressure data collected before and after the intervention were processed and analyzed with IBM SPSS Statistics for Windows, Version 20. The data, deviating from normal distribution according to the Kolmogorov-Smirnov test, underwent descriptive statistical analysis to calculate means, standard deviations, frequencies, and percentages. Differences in physiological measures between genders were examined using the Mann-Whitney U test, with a statistical significance set at p<0.05.

## Results

Our research encompassed 252 participants whose demographic details are outlined in Table 1. The composition was evenly split across genders, comprising 118 females (46.8%) and 134 males (53.2%). The age distribution spanned from 7 to 12 years with 95 participants aged seven (37.7%), 24 participants aged eight (9.5%), 46 participants aged nine (18%), 35 participants aged ten (13.9%), 19 participants aged eleven (7.5%), and 33 participants aged twelve (13.1%). This distribution underscores the adherence to the established selection criteria, affirming an equitable and uniform representation across both study groups. 

**Table 1:** Age and gender distribution of the study participants. N: Number of participants, %- Percentage

Demographic characteristics (N = 252)		N	%
Age (years)	7	95	37.7
	8	24	9.5
	9	46	18.3
	10	35	13.9
	11	19	7.5
	12	33	13.1
Gender	Male	134	53.2
	Female	118	46.8

The evaluation of the behavioral intervention on physiological measures shows significant outcomes. The experimental (EXP) group demonstrated a notable reduction in the post-intervention mean heart rate for both males (87.97 bpm) and females (89.81 bpm) (p=0.391), compared to an increase observed in the control group (males: 98.79 bpm, females: 102.13 bpm) (p=0.830). These changes, however, did not reveal statistically significant gender differences.

In terms of systolic blood pressure (SBP), there was a decrease in the EXP group after the intervention (males: 96.10 mmHg, females: 90.62 mmHg), while an increase was noted in the control group. A significant gender difference in SBP was found only in the post-intervention phase (p<0.05) within the EXP group (p = 0.007), with no significant disparities in the pre-intervention phase or within the control group at any evaluated time.

The mean diastolic blood pressure (DBP) also decreased from pre- to post-intervention in the EXP group (males: 73.07 mmHg, females: 72.17 mmHg) (p=0.746), while it increased in the control group. This fluctuation did not exhibit significant gender differences in either group at any phase of the study.

**Table 2:** Relationship between gender and body vitals. *p<0.05; The Mann-Whitney U test was used. N: Number of participants, Mean±SD: mean and standard deviation; EXP: Alpha wave entrainment group (N = 128): Male (N = 70) and Female (N = 58); CON: Control Group (N =124): Male (N = 64) and Female (N = 60).

Body vitals (N = 252)	Groups	Intervention	Gender	Mean±SD	Mann-Whitney U test value (p-value)
Heart Rate (beats/min)	EXP	Pre	Male	100.51±16.60	1708.500 (0.124)
			Female	104.41±18.63	
		Post	Male	87.97±11.56	1851.000 (0.391)
			Female	89.81±12.56	
	CON	Pre	Male	91.07±13.42	1905.000 (0.940)
			Female	95.01±19.13	
		Post	Male	98.79±14.22	1877.000 (0.830)
			Female	102.13±20.40	
Systolic Blood Pressure (mmHg)	EXP	Pre	Male	105.00±10.96	2000.500 (0.888)
			Female	103.00±12.42	
		Post	Male	96.10±9.62	1463.000 (0.007)*
			Female	90.62±10.68	
	CON	Pre	Male	105.07±12.70	1691.000 (0.252)
			Female	102.71±14.07	
		Post	Male	109.32±11.30	1822.000 (0.624)
			Female	109.20±17.95	
Diastolic Blood Pressure (mmHg)	EXP	Pre	Male	76.57±8.96	1645.500 (0.065)
			Female	79.89±11.63	
		Post	Male	73.07±9.26	1962.500 (0.746)
			Female	72.17±10.60	
	CON	Pre	Male	77.98±10.35	1770.000 (0.453)
			Female	76.31±10.52	
		Post	Male	79.81±11.24	1840.000 (0.689)
			Female	79.03±11.18	

## Discussion

The phenomenon of dental anxiety, particularly within the pediatric demographic, presents a significant challenge in dental care, necessitating interventions that extend beyond conventional pharmacological remedies [7]. This study embarked on an exploration of alpha wave entrainment (AWE), a non-pharmacological intervention, aiming to substantiate its efficacy in mitigating dental anxiety among pediatric patients [8-9]. With a meticulously designed methodological framework, the research encompassed a balanced cohort of 252 participants, ensuring a representation that aligns with established selection criteria. This study's methodological foundation was robust, encompassing a demographically balanced sample of 252 pediatric patients, ensuring a comprehensive investigation across genders (118 females and 134 males) and a broad age spectrum (7 to 12 years). This careful selection underscores our commitment to generating insights that are both generalizable and reflective of the diverse pediatric population we aim to serve [10-12].

The empirical evidence from our study highlights the significant potential of alpha wave entrainment (AWE) in mitigating dental anxiety through its impact on physiological markers. In the post-treatment phase, the intervention group exhibited a notable reduction in mean heart rates, with males and females achieving 87.97 bpm and 89.81 bpm, respectively. This marked decrease contrasts sharply with the control group's increases to 98.79 bpm for males and 102.13 bpm for females, illustrating AWE's effectiveness in inducing a state of relaxation. Such findings not only underscore AWE's capacity to harmonize cerebral wave frequencies with external stimuli but also align with the neurophysiological mechanisms of entrainment discussed by Will et al. and Garcia-Argibay et al. [1,12-13]. Their seminal work elucidates entrainment's role in promoting relaxation and well-being, suggesting its universal applicability across genders. This is particularly notable given the lack of statistically significant gender disparity (p>0.05) in our results, thereby affirming the broad efficacy of AWE in reducing dental anxiety among pediatric patients.

Subsequent analysis of the intervention's impact on systolic and diastolic blood pressure (SBP and DBP) elucidated significant trends that underscore the nuanced efficacy of alpha wave entrainment (AWE) in modulating physiological responses associated with anxiety. Within the intervention cohort, a marked reduction in SBP was observed (males: 96.10 mmHg, females: 90.62 mmHg), starkly contrasting with the control group's elevated SBP levels. This differential response, particularly pronounced post-intervention (p = 0.007), underscores a significant gender-specific variance in SBP outcomes. Such findings align with contemporary research, notably the study by Lopez-Diaz et al. and Padmanabhan et al., which highlights the complex physiological nuances inherent in stress and anxiety management across genders [2,14]. This insight also finds backing in the systematic review by Giani et al. on the efficacy of cognitive-behavioral therapy in treating separation anxiety [3,15-17].

The examination of diastolic blood pressure (DBP) metrics within the intervention group unveiled a subtle, though not statistically significant, downward trend across genders, contrasting with the outcomes observed in the control group, aligning with the research by Barlow (1960) and Bartley (1934) [5-6]. This modest reduction in DBP (p>0.05) serves not only as a reaffirmation of the potential of BWE in attenuating physiological markers indicative of anxiety but also aligns with the deeper neurophysiological mechanisms that underscore entrainment's efficacy. The seminal research conducted by Lane et al. and Lentz et al. provides a critical foundation for interpreting these physiological alterations, demonstrating an enhancement in EEG amplitude under the influence of both auditory and visual entrainment conditions [18-19]. This amplification in neural activity is indicative of AWE's capacity to synchronize cerebral wave frequencies with external stimuli, facilitating a state of relaxation and well-being.

The implications of these findings extend far beyond the immediate physiological effects, offering insights into the potential clinical applications of AWE within pediatric dentistry. Lane et al. and Lentz et al. have elucidated the synergistic impact of integrating auditory and visual stimuli, a strategy that maximizes the relaxation and anxiety mitigation effects of entrainment [7,18-19]. This synergy is particularly relevant in the pediatric dental context, where ensuring patient comfort and fostering cooperative behavior are of utmost importance [20]. The ability of AWE to induce a relaxed state without the need for pharmacological interventions presents a compelling alternative for managing dental anxiety, one that is grounded in neurophysiological principles and validated by empirical evidence [21-24].

Moreover, the nuanced understanding of the effects of AWE on DBP and its broader neurophysiological implications underscores the importance of adopting a holistic approach to patient care. By leveraging the synergistic potential of auditory and visual stimuli, AWE offers a non-invasive means of enhancing the dental care experience for pediatric patients, potentially improving clinical outcomes and patient satisfaction. The exploration of AWE's efficacy in reducing dental anxiety, particularly in light of its impact on physiological markers like DBP, is indicative of the evolving landscape of pediatric dental care, a landscape increasingly informed by interdisciplinary research and the integration of neuroscientific insights into clinical practice.

The exploration of gender-specific responses to AWE, particularly the significant variance in SBP response, adds a layer of complexity to the discourse [25]. This finding prompts a call for further investigation into the biological, psychological, and social determinants of gender differences in stress and anxiety responses. Understanding these nuances is crucial for tailoring AWE interventions to individual patient needs, ensuring optimal outcomes across the pediatric population. This approach is supported by extensive research, including the exploratory study on photic driving and altered states of consciousness by Glicksohn (1986) and the investigation into binaural beats' efficacy in reducing preoperative dental anxiety by Isik et al. [13,26].

The implications of our study extend beyond the immediate context of dental anxiety management. By demonstrating the efficacy of a non-pharmacological, universally applicable intervention like AWE, we pave the way for a broader re-evaluation of patient care strategies in pediatric dentistry. This approach, grounded in empirical evidence and neurophysiological theory, promotes a shift towards more holistic, patient-centered care modalities that prioritize patient well-being alongside procedural success [27].

While the study's findings are promising, certain limitations should be acknowledged to better contextualize the results. One potential limitation is the short-term assessment period which does not provide insight into the long-term effectiveness of AWE in managing dental anxiety, necessitating further longitudinal studies. Moreover, encouraging children to keep their eyes closed for a duration of 10 minutes can be challenging, particularly due to their naturally hyperactive nature [28]. Addressing this issue required a dedicated examiner to closely monitor and track the children's eye movements during the intervention.

Future research avenues are abundant, with a need to explore the long-term effects of AWE on dental anxiety, optimize intervention protocols for maximum efficacy, and further elucidate the mechanisms underlying gender-specific responses. Additionally, integrating AWE with other therapeutic modalities, such as cognitive-behavioral therapy, could offer synergistic benefits, harnessing the full potential of non-pharmacological interventions in managing pediatric dental anxiety.

## Conclusions

In conclusion, the application of alpha wave entrainment has been demonstrated to be effective in reducing pre-operative anxiety among pediatric dental patients. Additionally, the analysis revealed no significant gender differences in the modulation of anxiety levels when assessed using physiological parameters. Incorporating AWE into pediatric dentistry could significantly improve patient experiences and mark a move towards more compassionate, evidence-based treatments. As we explore dental anxiety further, AWE stands out as a promising approach for advancing patient-centered pediatric dental care.

## References

[1] Will U, Berg E (2007). Brain wave synchronization and entrainment to periodic acoustic stimuli. Neurosci Lett.

[2] Lopez-Diaz K, Henshaw J, Casson AJ (2021). Alpha entrainment drives pain relief using visual stimulation in a sample of chronic pain patients: a proof-of-concept controlled study. Neuroreport.

[3] Giani L, Caputi M, Forresi B, Michelini G, Scaini S (2022). Evaluation of cognitive-behavioral therapy efficacy in the treatment of separation anxiety disorder in childhood and adolescence: a systematic review of randomized controlled trials. Int J Cogn Ther.

[4] Joyce M, Siever D (2000). Audio-visual entrainment program as a treatment for behavior disorders in a school setting. J Neurother.

[5] Barlow JR (1960). Rhythmic activity induced by photic stimulation in relation to intrinsic alpha activity of the brain in man. Electroencephalogr Clin Neurophysiol.

[6] Bartley S Relation of intensity and duration of brief retinal stimulation by light to the electrical response of the optic cortex of the rabbit. Am J Physiol.1934.

[7] Shehani AF, Ponraj S, Ramar K, Samuel VA, Rajakumar S, Gayathri J (2023). Non-pharmacological behavior management techniques in pediatric dentistry: a bibliometric analysis. Cureus.

[8] Isik BK, Esen A, Büyükerkmen B, Kilinç A, Menziletoglu D (2017). Effectiveness of binaural beats in reducing preoperative dental anxiety. Br J Oral Maxillofac Surg.

[9] Budzynski T, Jordy J, Budzynski HK, Tang HY, Claypoole K (1999). Academic performance enhancement with photic stimulation and EDR feedback. J Neurother.

[10] Venham L, Bengston D, Cipes M (1977). Children's response to sequential dental visits. J Dent Res.

[11] Gao X, Cao H, Ming D (2014). Analysis of EEG activity in response to binaural beats with different frequencies. Int J Psychophysiol.

[12] Garcia-Argibay M, Santed MA, Reales JM (2019). Efficacy of binaural auditory beats in cognition, anxiety, and pain perception: a meta-analysis. Psychol Res.

[13] Glicksohn J (1986). Photic driving and altered states of consciousness: an exploratory study. Imagin Cogn Pers.

[14] Padmanabhan R, Hildreth AJ, Laws D (2005). A prospective, randomised, controlled study examining binaural beat audio and pre-operative anxiety in patients undergoing general anaesthesia for day case surgery. Anaesthesia.

[15] Jirakittayakorn N, Wongsawat Y (2017). Brain responses to a 6-Hz binaural beat: effects on general theta rhythm and frontal midline theta activity. Front Neurosci.

[16] Kasprzak C (2011). Influence of binaural beats on EEG signal. Acta Phys Polo A.

[17] Keihani A, Shirzhiyan Z, Farahi M (2018). Use of sine shaped high-frequency rhythmic visual stimuli patterns for SSVEP response analysis and fatigue rate evaluation in normal subjects. Front Hum Neurosci.

[18] Lane JD, Kasian SJ, Owens JE, Marsh GR (1998). Binaural auditory beats affect vigilance performance and mood. Physiol Behav.

[19] Lentz JJ, He Y, Townsend JT (2014). A new perspective on binaural integration using response time methodology: super capacity revealed in conditions of binaural masking release. Front Hum Neurosci.

[20] Marwah N, Prabhakar AR, Raju OS (2005). Music distraction - it’s efficacy in the management of anxious pediatric dental patients. J Indian Soc Pedod Prev Dent.

[21] Locker D (2003). Psychosocial consequences of dental fear and anxiety. Community Dent Oral Epidemiol.

[22] Menziletoglu D, Guler AY, Cayır T, Isik BK (2021). Binaural beats or 432 Hz music? Which method is more effective for reducing preoperative dental anxiety?. Med Oral Patol Oral Cir Bucal.

[23] Sappey-Marinier D, Calabrese G, Fein G, Hugg JW, Biggins C, Weiner MW (1992). Effect of photic stimulation on human visual cortex lactate and phosphates using 1H and 31P magnetic resonance spectroscopy. J Cereb Blood Flow Metab.

[24] Senani R, Bhaskar DR, Singh VK, Sharma RK (2016). Sinusoidal oscillators and waveform generators using modern electronic circuit building blocks.

[25] Horton MA (2019). Human factors in dentistry. Prim Dent J.

[26] Garcia-Argibay M, Santed MA, Reales JM (2019). Binaural auditory beats affect long-term memory. Psychol Res.

[27] Pratt H, Starr A, Michalewski HJ, Dimitrijevic A, Bleich N, Mittelman N (2010). A comparison of auditory evoked potentials to acoustic beats and to binaural beats. Hear Res.

[28] Rammsayer T, Netter P, Vogel WH (1993). A neurochemical model underlying differences in reaction times between introverts and extroverts. Pers Individ Dif.

